# YouTube as a Source of Patient Information for Cerebral Palsy

**DOI:** 10.3390/healthcare13131492

**Published:** 2025-06-23

**Authors:** Julia Stelmach, Jakub Rychlik, Marta Zawadzka, Maria Mazurkiewicz-Bełdzińska

**Affiliations:** Department of Developmental Neurology, Medical University of Gdansk, 80-952 Gdansk, Poland

**Keywords:** cerebral palsy, YouTube, DISCERN, social media

## Abstract

**Background/objectives:** Social media has significantly enhanced access to medical knowledge by enabling rapid information sharing. With YouTube being the second-most popular website, we intended to evaluate the quality of its content as a source of information for patients and relatives for information about cerebral palsy. **Methods:** The first 30 videos for search terms “Cerebral palsy”, “Spastic cerebral palsy”, “Dyskinetic cerebral palsy”, “Worster-Drought syndrome”, and “Ataxic cerebral palsy” were selected for inquiry. Out of 150 films, a total of 83 were assessed with a mixed method approach by two independent raters utilizing evidence-based quality scales such as Quality Criteria for Consumer Health Information (DISCERN), the Journal of the American Medical Association instrument (JAMA), and the Global Quality Score (GQS). Furthermore, audience engagement was analyzed, and the Video Power Index (VPI) was calculated for each video. **Results:** The mean total DISCERN score excluding the final question (subjective assessment of the video) was 30.5 ± 8.7 (out of 75 points), implying that the quality of the videos was poor. The global JAMA score was 2.36 ± 0.57 between the raters. The mean GQS score reached 2.57 ± 0.78. The videos had statistically higher DISCERN scores when they included treatment options, risk factors, anatomy, definition, information for doctors, epidemiology, doctor as a speaker, and patient experience. **Conclusions:** YouTube seems to be a poor source of information for patients and relatives on cerebral palsy. The analysis can contribute to creating more engaging, holistic, and informative videos regarding this topic.

## 1. Introduction

Cerebral palsy is the most common childhood physical disability, affecting approximately 2 to 3 per 1000 live births [1]. It results from early brain injuries that impair movement, posture, and balance [2], and may also cause muscle stiffness, involuntary movements, speech difficulties, and, in some cases, cognitive delays. CP affects children of all backgrounds and varies widely in severity. Although incurable, early and multidisciplinary therapies—such as physical, occupational, and speech therapy—can greatly improve quality of life [3]. Access to reliable treatment information is crucial for families and caregivers. Key sources include healthcare providers, medical websites (e.g., CDC and NHS), patient organizations, and online support groups.

YouTube, the world’s second-most popular website, is an increasingly important source of health information for people without medical backgrounds [4]. Therefore, maintaining high-quality content is crucial to ensure that reliable medical knowledge is available to patients and their families. Previous studies have evaluated the reliability of YouTube videos on topics such as hydrocephalus, stroke, migraine, fibromyalgia, and selective dorsal rhizotomy [5,6,7,8,9]. A recent evaluation (6 March 2022) also assessed cerebral palsy, but it was limited to videos in Brazilian Portuguese [10], a language spoken by only 2.5% of the global population, despite 18.1% of people speaking English, where much more content on cerebral palsy exists [11]. Cerebral palsy has also previously been analyzed in two YouTube-based studies; however, the absence of standardized evaluation scales both limited the credibility and consistency of their results.

YouTube videos have been shown to influence patient decision-making both positively, by offering better-informed choices, and negatively, by presenting biased or misleading information that could harm the patient–doctor relationship [12].

This study aims to assess the credibility of YouTube videos on cerebral palsy that patients and families are accessing. We aim to identify the features and characteristics that enhance audience engagement, particularly regarding the understanding of cerebral palsy risk factors, to help content creators develop more engaging and informative videos in the future.

## 2. Materials and Methods

### 2.1. Search Strategy and Data Collection

YouTube was used to collect data on cerebral palsy using five keywords: “Cerebral palsy”, “Spastic cerebral palsy”, “Dyskinetic cerebral palsy”, “Worster-Drought syndrome”, and “Ataxic cerebral palsy”. They were chosen based on being commonly used by both patients and medical professionals to describe this condition. The first 30 videos for each keyword were assessed as 90% of users do not search beyond the 30th result [13]. Videos were accessed via Google Chrome in incognito mode to avoid personal recommendations and evaluated independently by two trained medical students (J.S. and J.R.). The DISCERN, JAMA, and GQS scoring systems were used to assess video quality. All data was publicly available so no special permission from YouTube was required.

### 2.2. Inclusion and Exclusion Criteria

A total of 150 videos (30 for each keyword) were initially recorded. Duplicates, non-English videos, and irrelevant content were excluded.

### 2.3. Extracted Variables

Quantitative data was extracted using the extension “vidIQ Vision for Youtube” for Google Chrome (browser version 3.105.0 for Google Chrome) for each video. The following data was extracted: words spoken per minute, duration, upload date, the number of comments, view count, video description word count, channel mean daily subscribers, video description link count, channel mean daily views, video referrers, and channel country of origin. The source of the upload was also analyzed and aligned into categories: an educational channel, a news channel, a hospital, a health organization, a physician, a patient, and miscellaneous (when the upload was unfitted in other criteria).

Qualitative data collected for each video included symptoms of cerebral palsy, risk factors, information on when to contact a doctor, anatomy, videos for doctors, definition, treatment options, etiology, epidemiology, animation, diagram, speaker doctor, and patient experience.

### 2.4. Scoring Systems

Two raters—medical students trained in using three different scoring systems: DISCERN [14] (Quality Criteria for Consumer Health Information), GQS (Global Quality Score), and JAMA [15] (Journal of the American Medical Association)—independently evaluated the quality of the videos. A mixed method approach was appropriate for this study as it allowed for both objective measurement of information quality using quantitative tools (e.g., the DISCERN instrument) and deeper insight into user perceptions and experiences through qualitative analysis. This combination provided a more comprehensive and nuanced understanding of the health information evaluated. The DISCERN instrument is a validated tool designed to assess the quality and reliability of written consumer health information, particularly regarding treatment options. It consists of 16 items, each rated on a five-point scale, evaluating key aspects such as clarity, relevance, transparency, and balance. The items are grouped into three sections: reliability (items 1–8), quality of information on treatment choices (items 9–15), and a unique overall quality rating (item 16). The final item provides a holistic assessment of the publication’s overall usefulness as a health information resource. DISCERN is widely used by healthcare professionals and patients to identify trustworthy, comprehensive, and unbiased health information.

Interpretation of the total DISCERN score has been previously established in the literature as excellent (63–75 points), good (51–62 points), fair (39–50 points), poor (27–38 points), or very poor (16–26) [16]. The video and the video description were taken into account for the assessment. The date that the video was uploaded may differ from the date the content was actually produced; therefore, when assessing question 7 (which addresses when the information was produced and reported), this distinction was taken into account.

The JAMA scoring system is a 4-point scale, giving one point for including each of four criteria: authorship, attribution, disclosure, and currency. The GQS score ranges from 0 to 5 points (extending from “poor” to “moderate” to “excellent”), evaluating the quality and flow of the video.

### 2.5. Audience Engagement

To evaluate the popularity of the videos, average daily views [total views/days since upload], Video Power Index (VPI) [(like × 100/(like + dislike)) × (views/day)/100], and like ratio [(likes/likes + dislikes) × 100] were calculated for each video. Correlations were examined between audience engagement metrics—average daily views, VPI, like ratio, and number of comments—and video quality indicators, including GQS, JAMA, and DISCERN scores, to evaluate the impact of content quality on viewer engagement.

### 2.6. Statistical Methods

Where applicable the mean, range, and standard deviation are marked as mean ± standard deviation (range). *p* < 0.05 was acknowledged as significant. The Shapiro–Wilk test was used to verify normality; the Mann–Whitney U test was applied to search for differences between categorical variables, and linear bivariate correlations were calculated with the Pearson correlation coefficient. Intraclass correlation coefficients were adopted to compare the consistency between raters. Canva (Canva Pty Ltd., Sydney, Australia) was used for illustrations. For statistical analysis, JASP (JASP TEAM, Version 0.18.3) was used.

## 3. Results

### 3.1. Video Contents

**Figure 1:**
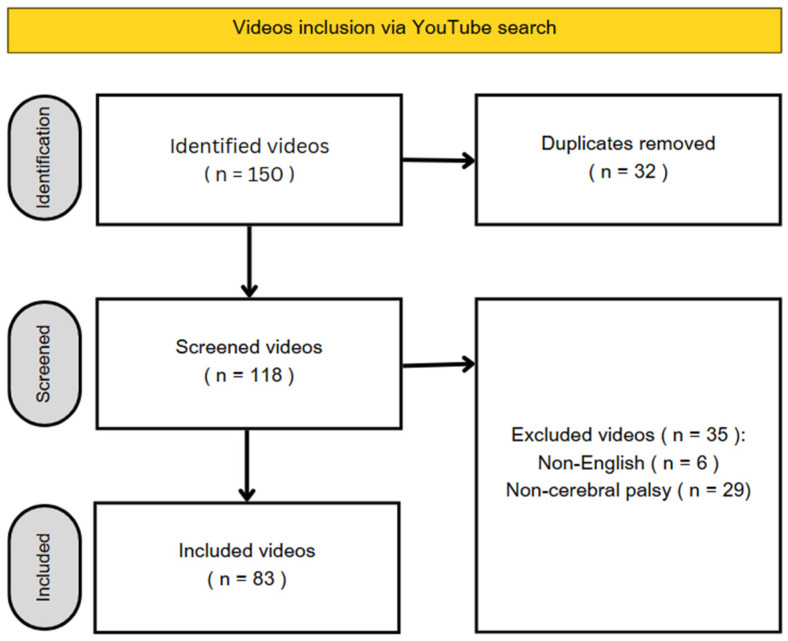
Video study inclusion.

After applying the exclusion criteria, 83 out of 150 videos were analyzed (Figure 1).

Table 1 shows that almost every video 74 (89.2%) included symptoms and only just over half 46 (55.4%) featured treatment options for cerebral palsy. In merely 33 (39.8%), the speaker was a doctor, and only 14 (16.9%) discussed risk factors.

### 3.2. Video Upload Sources

**Figure 2:**
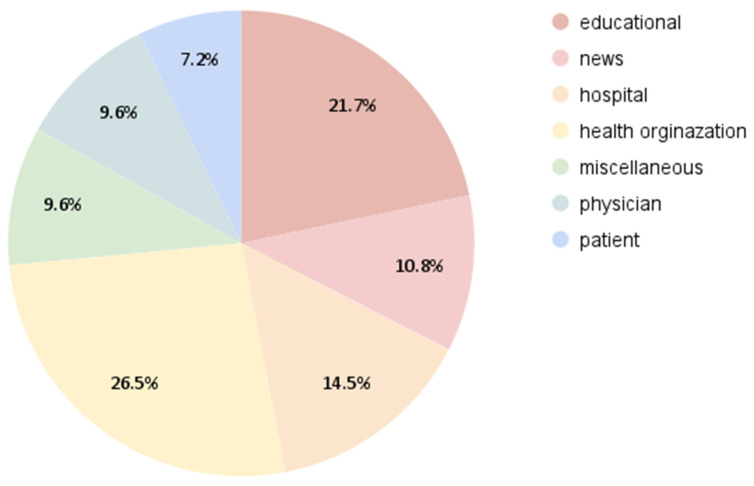
Upload sources.

Figure 2 shows the sources of the videos uploaded. Most were published by health organizations 22 (26.5%), educational channels 18 (21.7%), and hospitals 12 (14.5%). News channels uploaded only nine (10.8%), physicians uploaded eight (9.6%), and patients uploaded six (7.2%).

### 3.3. Statistics of Video

Table 2 shows the mean and range for each of the quantitative metrics measured; they are as follows: view count, 86,676 (range: 333–1,073,744); number of comments, 30 (0–398); number of likes, 826 (0–25,934); number of dislikes, 20 (0–271); average daily views, 44 (0–431); duration, 273 (16–2372) s; video description word count, 92 (0–507); video description link count, 2 (0–48); and days since upload, 2071 (259–5505).

To assess the average popularity of the channels hosting the videos, the following metrics were used: subscribers 550,629 (0–15,674,610), daily views 153,579 (1–290,000), and daily subscribers 244 (0–3333).

### 3.4. Video Quality Evaluation

The total mean DISCERN score given by the first student rater and second student rater for the first 15 questions was 30.5 ± 8.7 (16–62); this is regarded as a poor score (the ‘poor’ scoring range being from 27 to 38).

The two raters had DISCERN scores of 29.9 ± 8.8 (16–59) and 31.1 ± 8.8 (19–62) correspondingly. The intraclass correlation coefficient (ICC) for absolute agreement between the two raters using the DISCERN instrument was 0.946 (95% CI: 0.918 to 0.965), indicating a high level of inter-rater reliability.

The entire score between the two raters for question 16 of DISCERN (which requires a global evaluation of the entire video) was 1.55 ± 0.19 (1–5). Raters had mean scores of 1.69 (1–5) and 1.42 (1–5) accordingly.

**Figure 3:**
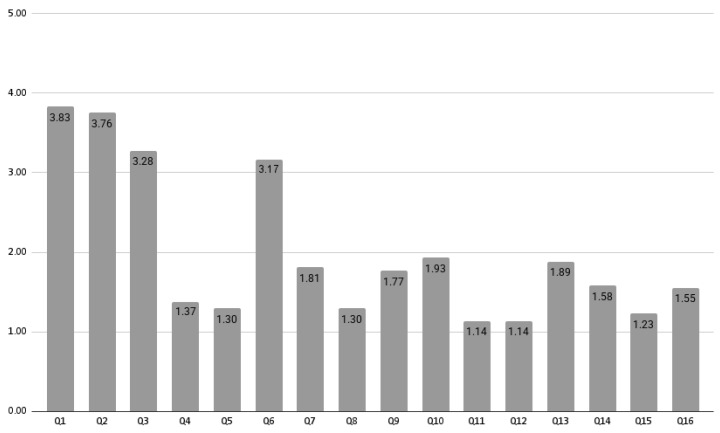
Mean DISCERN scores for each question.

The mean DISCERN scores for each of the 16 questions are illustrated in Figure 3. Questions 1 to 3 and question 6 received the highest ratings, all scoring above three in descending order. These questions addressed whether the aims are clear, if the resource achieves its aim, whether it is relevant, and if it is balanced and unbiased. In contrast, questions 11 and 12 received the lowest ratings, both scoring below 1.2. These questions focused on the consequences of not receiving treatment and the potential impact of treatment on quality of life.

The global JAMA score between the first rater and second rater was 2.36 ± 0.57 (1–4). Raters had JAMA scores of 2.31 ± 0.52 (1–4) and 2.40 ± 0.62 (1–4) accordingly. The intraclass correlation coefficient for absolute agreement for the JAMA score between the two raters was 0.859 (95 % CI 0.789 to 0.906).

The two raters had a mean GQS score of 2.57 ± 0.78 (1–5). The mean score per individual rater was 2.54 ± 0.79 (1–5) and 2.60 ± 0.78 (1–5), respectively. The intraclass correlation coefficient for absolute agreement for the GQS between the two raters was 0.913 (95 % CI 0.869 to 0.943).

### 3.5. Video Quality Correlations

We found a significant positive correlation between the DISCERN score and videos that included the following elements: treatment options (s) (*p* < 0.001), risk factors (*p* = 0.022), anatomy (*p* = 0.022), definition (*p* = 0.044), time (*p* < 0.001), information for doctors (*p* = 0.001), epidemiology (*p* = 0.045), doctor as a speaker (*p* = 0.003), patient experience (*p* = 0.007), and made by patients (*p* = 0.003). The other variables did not influence the DISCERN score.

### 3.6. Audience Engagement Analysis

Videos had a higher VPI score when they included video description word count (*p* < 0.001), video description link count (*p* < 0.001), anatomy (*p* = 0.034), information for doctors (*p* = 0.020), and animation (*p* = 0.015). The rest of the analyzed factors did not affect the VPI score.

### 3.7. Top Quality Videos

Table 3 represents the top five highest-quality cerebral palsy videos based on DISCERN criteria. Only two of them are good quality (from 51 to 62 DISCERN points) and three are average quality (from 39 to 50 DISCERN points). None of them reached excellent quality (63–75 DISCERN points).

## 4. Discussion

### 4.1. Analysis of Quality

We found that the quality and reliability of YouTube videos on cerebral palsy were generally poor, with a mean DISCERN score of 30.5 out of a possible 75. This suggests that the medical information conveyed by YouTube seems to be incomplete and limited. Our findings are novel as our paper is the first to perform a content quality evaluation and audience engagement analysis. A higher DISCERN score was associated with elements such as anatomy, time, information for doctors, epidemiology, doctor as a speaker, patient experience, and made by patients. Allegedly it may be connected with a sense of quality and reliability.

Barely 2 videos out of 83 were qualified as “good” when it comes to DISCERN scale evaluation. The majority of videos fail to indicate the source and date of the reported information. Above 55% mention treatment options; however, not all of them describe how each treatment works, what the benefits are, and the possible effects of therapy choices on overall quality of life. Even less comment was provided on the risks of treatment and what could happen if no treatment was applied. Furthermore, some did not describe if there are any other possible therapy choices. All of the mentioned factors influence scoring in DISCERN.

### 4.2. Suggestions for Quality Improvement

The low DISCERN scores reflect insufficient coverage of treatment options. While the JAMA score showed that videos frequently list authors and upload dates, they rarely included references or disclosed ownership of the material. Many videos lacked Supplementary Information to guide patients seeking further knowledge. Greater focus on treatment options would improve video quality as it is a key area for patient decision-making. Fulfilling DISCERN, JAMA, and GQS criteria would lead to more balanced and informative videos, enhancing public trust. Additionally, including elements such as treatment options, risk factors, anatomy, definition, timing, information for doctors, patient experience, and epidemiology—which have been shown in our results to correlate with a higher DISCERN score—may improve the quality and informativeness of the videos. It is worth adding that nearly every video insufficiently discussed the consequences of lack of treatment and its impact on quality of life. Both of these aspects are essential for informed decision-making, especially for chronic conditions like cerebral palsy where understanding the risks of non-treatment and the lived impact of interventions is crucial. Addressing this gap presents a clear opportunity for creators to improve educational value by incorporating real-life scenarios, patient experiences, and outcome-based discussions.

### 4.3. Audience Engagement Evaluation

Including anatomy in videos led to more views and comments, suggesting that audiences appreciate a clear understanding of cerebral palsy from an anatomical perspective. Animations also boosted engagement, likely due to their ability to simplify complex concepts. Diagrams, risk factors, and information on epidemiology and etiology increased audience interaction across views, likes, and comments. These elements create an effective formula for presenting medical information. Additionally, videos narrated by doctors or featuring patient experiences saw significantly higher engagement, indicating that viewers trust content delivered by professionals and are also interested in hearing from patients.

Interestingly, no correlation was found between audience engagement metrics and quality scores (GQS, JAMA, and DISCERN), suggesting that the attribution of sources and ownership of data are not a primary concern for patients and their families. Moreover, better quality of material is not necessarily connected with higher audience engagement. The number of likes may reflect entertainment value rather than the accuracy or reliability of the health information presented. Viewers often lack the expertise to assess content quality, leading to popularity that does not necessarily align with informational value. Surprisingly, video description word count and video link word count are associated with a higher VPI score; supposedly a higher number of words may seem more sophisticated.

### 4.4. Context

Cerebral palsy is the most common childhood physical disorder, affecting 2 to 3 out of every 1000 children, but only a few studies have yet assessed the accuracy and trustworthiness of YouTube videos on this topic in English. Previous studies on the quality of YouTube videos related to neurological conditions like aneurysms, meningiomas, and stroke treatment also reported poor or fair quality [5,17,18]. Our study suggests that the quality of cerebral palsy videos could improve by including more detailed information about treatment options, their benefits and risks, and their impact on patients’ quality of life. Enhancing content with such information may help patients make more informed decisions, ultimately contributing to better treatment adherence and improved quality of life for individuals with cerebral palsy.

Although cerebral palsy has been studied in YouTube videos twice before, those studies lacked standardized evaluation scales, limiting their reliability. While YouTube is a popular source for medical information, other platforms, such as Google and online patient forums, are also frequently used to understand medical conditions. However, studies, such as one on migraine [19], show that many online resources are too complex for the average patient. The quality of medical content is further compromised by competing advertisements and promotional material.

Moreover, many patients consult online forums for answers, especially when facing new diagnoses. These forums are often linked to anxiety and confusion, especially when patients struggle to make informed decisions about treatments. Additionally, misleading information is common on YouTube despite the availability of credible sources [20]. None of the videos we analyzed received an “excellent” quality score, and only two videos were rated as “good” according to the DISCERN scale. Therefore, it is crucial to establish quality standards for health-related content on YouTube in accordance with the findings.

Patients searching for cerebral palsy videos often focus on surgical treatments [21]. However, cerebral palsy requires multi-faceted treatment, including physical therapy, medication, and sometimes surgery. Our analysis found that information on treatment was particularly lacking, which could influence patients’ decisions regarding surgeries, such as selective dorsal rhizotomy or orthopedic procedures. Selective dorsal rhizotomy can improve quality of life but requires extensive follow-up therapy, and orthopedic surgeries need to be assessed on an individual basis for potential benefits and risks.

Several quality evaluations of YouTube videos have been conducted by physicians [17,18,22], but the DISCERN tool is designed to be usable without specialist knowledge [14]. In our study, medical students assessed the videos to demonstrate that specialized knowledge was not necessary to evaluate the quality of these videos.

### 4.5. Limitations

This cross-sectional study only included 150 videos, which may limit the sensitivity of the results. However, since 90% of YouTube users do not go beyond the first 30 search results [13], we considered 30 videos per keyword a sufficient sample size. Algorithmic bias could influence our study since YouTube’s searches are affected by users’ location, search history, and engagement metrics [23], potentially missing high-quality but less-viewed content and limiting reproducibility. In our study only English videos were included, which excludes potentially high-quality videos in other languages. Our audience engagement analysis may suffer from selection bias as not all viewers who like or dislike videos express their opinions. This limitation is common in YouTube studies and not specific to our research.

The DISCERN, JAMA, and GQS instruments used in this study are validated and widely applied in social media quality evaluations [5,6,7,8,9,17,18,22,24,25,26], and the intraclass correlation between raters was excellent, indicating dependable results. Although some may argue that medical students are less reliable than healthcare professionals for video rating, the DISCERN tool was specifically designed to be independent of specialist knowledge [14]. This was confirmed in a separate evaluation of the tool, which showed that both patients and healthcare professionals can use it effectively to assess information quality. Future studies could compare assessments by healthcare providers and medical students.

It is important to note that YouTube videos are not intended to replace professional medical advice but to educate the public, including cerebral palsy patients and their families, and guide them toward seeking specialist care. Our study only analyzed videos in English, so future research should explore content quality in other languages. Additionally, 24% of the videos analyzed did not disclose the country of origin, limiting the scope of our findings.

### 4.6. Future Directions

A study similar to this one should be repeated in a few years to assess the difference in the quality of YouTube videos since new videos are uploaded every day. Furthermore, the results of YouTube database searches change over time due to mechanisms based on relevancy. Moreover, further research is needed in the form of surveys to determine whether viewers feel they are receiving accurate and useful information from YouTube, as well as to identify the sources they reference and consider trustworthy. Additionally, conducting similar studies in languages other than English would be valuable to evaluate the reliability of medical information on YouTube for non-English-speaking audiences. Although English is the most widely used language globally, over 80% of the world’s population may be excluded from fully accessing and benefiting from online video content due to language barriers. This highlights the importance of analyzing health-related videos in non-English languages to ensure equitable access to reliable information.

## 5. Conclusions

As the second-most visited website worldwide, YouTube plays a significant role in how the general public accesses health-related information. It is of considerable importance for physicians and medical institutions to ensure that the available data is of good quality and contains valid data. The JAMA benchmarks, DISCERN instrument, and GQS might be valuable guides for increasing the quality of the recording. YouTube offers limited reliable health-related information for patients about cerebral palsy, highlighting a lack of quality content available to viewers. In our study, we have identified and listed the highest-quality videos to help physicians guide their patients toward trustworthy resources.

## Figures and Tables

**Table 1:** Information found in the analyzed material.

Criterion	Count	Percentage
Symptoms	74	89.16
Risk factors	14	16.87
When to contact doctor	2	2.41
Anatomy	20	24.10
Definition	22	26.51
Treatment options	46	55.42
Etiology	30	36.14
Epidemiology	11	13.25
Animation	27	32.53
Diagram	2	2.41
Doctor speaking	33	39.76
Patient experience	57	68.67
For doctors	30	36.14
Popular science	53	63.86

**Table 2:** Statistics of videos.

Video Metrics	Mean	Range
View count	86,676	333–1,073,744
Number of comments	30	0–398
Number of likes	826	0–25,934
Number of dislikes	20	0–271
Average daily views	44	0–431
Duration (s)	273	16–2372
Video description word count	92	0–507
Video description link count	2	0–48
Days since upload	2071	259–5505
Subscribers	550,629	0–15,674,610
Daily views	153,579	1–2,900,000
Daily subscribers	244	0–3333

**Table 3:** Top five highest-quality videos.

DISCERN	JAMA	Title	Uploader
60.5	3	Treating Spasticity in Cerebral Palsy	Utah Neuro Rehabilitation
54.5	3	Cerebral Palsy (CP)|Etiology, Spastic, Dyskinetic, Ataxic, Diagnosis, and Treatment	Sqadia.com
49.5	4	Spasticity Treatment for Cerebral Palsy|Gillette Children’s	Gilette Children’s
48.5	4	Dyskinetic Cerebral Palsy: Efficacy of Oral Pharmacological Treatments|Riccardo Masson|DMCN	DMCNvideos
48	2	Cerebral Palsy: Etiology, Pathophysiology, Complications, Treatment	Alila Medical Media

## Data Availability

The data presented in this study are available on request from the corresponding author.

## Abbreviations

JAMA	Journal of the American Medical Association instrument
GQS	Global Quality Score
VPI	Video Power Index
CI	Confidence interval
s	Second(s)
